
JOURNAL INFORMATION
==============================
NLM Title Abbreviation: Arch Public Health
Journal ID: Arch Public Health
NLM Journal ID: 9208826

Archives of Public Health

ISSN: 0778-7367
EISSN: 2049-3258
Publisher: BMC

ARTICLE INFORMATION
==============================
PMCID: PMC6397331
DOI: 10.1186/2049-3258-72-S1-P2
Article ID: 5118
Article version: 1
Subjects: Poster Presentation

Abstract Withdrawn

Electronic publication date: 2014 Jun 6
Publication date: 2014
Volume: 72
Issue: Suppl 1
Issue sponsor: The publication of this supplement has been funded by FEVIA and FOD Health, food chain safety and environment and embedded in the National Food and Health Plan. The Supplement Editors declare that they have no competing interests.
Electronic Location ID: P2
Copyright: © Michels et al; licensee BioMed Central Ltd. 2014
License: This article is published under license to BioMed Central Ltd. This is an Open Access article distributed under the terms of the Creative Commons Attribution License (http://creativecommons.org/licenses/by/4.0), which permits unrestricted use, distribution, and reproduction in any medium, provided the original work is properly cited. The Creative Commons Public Domain Dedication waiver (http://creativecommons.org/publicdomain/zero/1.0/) applies to the data made available in this article, unless otherwise stated.
License URL: https://creativecommons.org/licenses/by/4.0/

Keywords: Public Health, Open Access, Public Domain, Original Work, Poster Presentation
Conference name: Genes and nutrition, is personalised nutrition the next realistic step?
Conference location: Brussels, Belgium
Conference date: 25 April 2014
issue-copyright-statement: © The Author(s) 2014

==============================
| PublisherInfo | | |
|---|---|---|
| PublisherName | : | BioMed Central |
| PublisherLocation | : | London |
| PublisherImprintName | : | BioMed Central |

Christophe Matthys

Carl Lachat

Johan Van Camp

Kristine Verbeke

Nathalie Delzenne

Patrick Kolsteren

Stefaan De Henauw

### Abstract Withdrawn

| ArticleInfo | | |
|---|---|---|
| ArticleID | : | 5118 |
| ArticleDOI | : | 10.1186/2049-3258-72-S1-P2 |
| ArticleCitationID | : | P2 |
| ArticleSequenceNumber | : | 23 |
| ArticleCategory | : | Poster presentation |
| ArticleFirstPage | : | 1 |
| ArticleLastPage | : | 1 |
| ArticleHistory | : | RegistrationDate : 2014–6–6<br>OnlineDate : 2014–6–6 |
| ArticleCopyright | : | Michels et al; licensee BioMed Central Ltd.2014<br>This article is published under license to BioMed Central Ltd. This is an Open Access article distributed under the terms of the Creative Commons Attribution License (http://creativecommons.org/licenses/by/4.0), which permits unrestricted use, distribution, and reproduction in any medium, provided the original work is properly cited. The Creative Commons Public Domain Dedication waiver (http://creativecommons.org/publicdomain/zero/1.0/) applies to the data made available in this article, unless otherwise stated. |
| ArticleGrants | : | |
| ArticleContext | : | 136907272S1S1 |

This PDF file was created after Publication.
